# Greenhouse and field evaluation of bacterial endophytes from *Cannabis sativa* and *Chelidonium majus* reveals beneficial stage-dependent growth and yield responses in common bean

**DOI:** 10.3389/fpls.2026.1922467

**Published:** 2026-08-27

**Authors:** Mohammad Jamil Kaddoura, Laura Amaya-Quiroz, Jamil Samsatly, Hacene Meglouli, Saji George

**Affiliations:** 1 Department of Food Science and Agricultural Chemistry, McGill University, Montreal, QC, Canada; 2 Biosun Solutions, Chambly, QC, Canada

**Keywords:** Bacillus, bacterial endophyte, biostimulant, Phaseolus vulgaris, plant-growth-promotion, plant-microbe interactions, pseudomonas, rhizosphere microbiome

## Abstract

Bacterial endophytes are increasingly recognized for their ability to enhance plant growth and productivity through multiple physiological and biochemical mechanisms. However, how these responses are coordinated across plant developmental stages and translate into final crop performance remains unclear. This study evaluated previously characterized bacterial endophytes belonging to *Bacillus subtilis* (BS-114, BS-120) and *Pseudomonas wadenswilerensis* (PPW-26) as plant growth-promoting agents in yellow bean under *in vitro*, greenhouse, and field conditions. Bacterial treatments enhanced early developmental responses relative to the non-inoculated control (NC), with BS-114 increasing germination (+19.1%) and seedling total root length (+71.3%), while PPW-26 increased root biomass (+61.7%). Under greenhouse conditions, BS-114 improved reproductive development, increasing bud count (+47.4%), immature pod count (+60.2%), and pod fresh mass (+42.1%). Cell-free extracts reproduced several of these beneficial effects, with the BS-114 cell-free extract (BS-114E) producing the highest cumulative pod fresh mass (+94.2%), suggesting the contribution of extracellular components to reproductive performance. Physiological and gene-expression analyses further indicated that treatment responses were not attributable to a single pathway. Among the measured gas-exchange parameters, photosynthetic rate increased (+175.8%) and stomatal conductance increased (+306.9%). Gene expression analyses showed treatment-specific upregulation of selected genes, with N-fixation-related expression (*nifH*) reaching +5.62 log_2_FC (49.31×) with the BS-114 live-cell treatment (BS-114L), while hormone-related responses were characterized by coordinated upregulation of auxin signaling-related expression +5.24 log_2_FC (37.89×) and cytokinin-related expression patterns consistent with increased biosynthesis and reduced degradation. Field validation showed that all treatments increased yield relative to NC, with the BS-114+BS-120 consortium achieving a +32.65% increase, which was comparable to NPK (+37.76%), despite limited shifts in soil microbial diversity and moderate variation in soil nutrients. Together, these findings indicate that the evaluated bacterial treatments improved plant performance through coordinated responses across multiple biological levels and plant developmental stages, supporting their application as plant growth-promoting agents under controlled and field conditions.

## 1 Introduction

Agricultural landscapes have continuously been shaped by major transformations in farming systems. The Green Revolution marked a major shift toward input-intensive systems and consequently led to substantial increases in agricultural productivity. Agrochemicals, which once played a central role in this transition, now threaten the long-term stability of food production systems through their substantial contribution to environmental pollution, soil degradation, and disruption of soil microbial communities (Meena et al., 2020). The increasing need to meet global food demand within the constraints of environmental sustainability, together with regulatory and market pressure for safer and cleaner agricultural inputs, has intensified the search for more sustainable crop production strategies.

In response, biological approaches have gained increasing attention as alternatives to conventional inputs. Microbial-based bioinoculants, particularly plant growth-promoting bacteria (PGPBs), have emerged as important tools for enhancing crop performance and resilience. Among PGPBs, root- and rhizosphere-associated bacteria, commonly referred to as plant growth-promoting rhizobacteria (PGPRs), include genera such as *Bacillus* and *Pseudomonas*, which are frequently associated with beneficial effects on plant development, physiological activity, and soil nutrient dynamics (Tariq et al., 2025; Wang et al., 2025). Some members of these root-associated groups can also colonize internal plant tissues as bacterial endophytes. The ability of beneficial bacterial endophytes to closely associate with the host plant and influence its functions makes them promising sources of effective bioinoculants. However, these endophytes are comparatively less understood, as a multitude of host- and environment-related factors influence their establishment, performance, and functional traits. Among these factors, host plant identity has been recognized as an important determinant of endophytic community composition. Scientific evidence supports a correlation between the host plant’s bioactive profile and its associated endophytic microbiota. Consequently, endophytes isolated from plants rich in metabolites, such as *Cannabis sativa* L. or *Chelidonium majus* L., represent promising sources for the development of effective multifunctional bioinoculants (Ahmed and Hijri, 2021; Stasińska-Jakubas et al., 2025).

To evaluate these bacterial endophytes in an agronomically relevant crop system, common bean (*Phaseolus vulgaris* L.), a widely cultivated and economically important legume crop, was selected as the model crop for this study. Its responsiveness to nutrient and microbial inputs, ability to form symbiotic associations, and measurable phenotypic responses across well-defined plant developmental stages make it suitable for evaluating biological inputs (Castro-Guerrero et al., 2016). Common bean has been widely used to assess responses to beneficial bacteria, with previous studies reporting PGPR-associated improvements in germination, canopy growth, root architecture, and physiological performance (Figueiredo et al., 2008; Remans et al., 2008; Meza et al., 2022). However, these responses vary widely across crops, systems, and biological levels, and depend on bacterial strain, host species, and environmental conditions, making it difficult to establish a clear mechanistic understanding (Kuzmicheva et al., 2017; Zhao et al., 2023). This limitation is important since most studies on beneficial bacteria have focused on PGPRs, whereas bacterial endophytes remain comparatively less studied. Beyond this, existing work on beneficial bacteria often examines plant responses at individual developmental stages or under controlled conditions, rather than through integrated evaluations across plant development and field conditions.

Understanding plant–microbe interactions under these constraints requires moving beyond individually assessed parameters and isolated experimental settings. Accordingly, this work builds on prior phenotypic and genotypic characterization of bacterial endophytes isolated from *C. sativa* and *C. majus*, which identified promising strains with traits associated with plant growth-promotion and biostimulant potential (Kaddoura et al., 2026). The current study extends their evaluation to plant-level validation in common bean and provides an integrated assessment across different biological and agronomic scales. Crop responses to the treatments are assessed across developmental stages from germination and seedling establishment through vegetative and reproductive development to final yield. Moreover, the study compares crop responses across different bacterial strains, formulations, and combinations with reduced fertilizer inputs. The work further integrates phenotypic, physiological, gene-expression, soil nutrient, and rhizosphere microbial responses and extends treatment assessment from controlled laboratory and greenhouse experiments to agronomic field conditions. Overall, this integrated approach provides a more complete basis for understanding bacterial treatment performance, crop responses, and the factors that may influence the development of effective and reliable microbial-based biostimulants.

## 2 Materials and methods

### 2.1 Bacterial strains and source

Bacterial strains used in this study were obtained from the culture collection of Dr. Saji George (S.A.F.E-Nano lab, McGill University, QC, Canada). The strains are bacterial endophytes originally isolated from *Cannabis sativa* L. and *Chelidonium majus* L. and were previously characterized for plant growth-promoting traits, abiotic stress tolerance, and fungal biocontrol activity (Kaddoura et al., 2026). Genome assemblies of *Bacillus subtilis* strain 114 (BS-114 accession: GCA_054481215.1), *B. subtilis* strain 120 (BS-120: GCA_054481115.1) and *Pseudomonas wadenswilerensis* strain 26 (PPW-26 accession: GCA_054481295.1) were deposited under BioProject PRJNA1379652 and are available through DDBJ/ENA/GenBank databases (Amaya-Quiroz et al., 2026).

### 2.2 Preparation of bacterial cell suspensions and cell-free extracts

Fresh bacterial suspensions were prepared from single colonies obtained from pure cultures streaked onto Luria-Bertani agar (LBA: tryptone, 10 g; NaCl, 10 g; yeast, 5 g; agar, 15 g; pH 7.0). A single bacterial colony of each strain was grown overnight (180 rpm, 30 °C) in LB broth to reach an adequate cell concentration (1×10^8^ CFU.mL^-1^). Unfractionated bacterial suspensions (UBS) were obtained directly from these overnight cultures. The remaining suspensions were then centrifuged (10, 000 rpm, 15 min, 10 °C). The resulting supernatants were collected as cell-free bacterial extracts (CFE), and the pellets were washed twice with phosphate-buffered saline (PBS: KH_2_PO_4_, 1.8 mM; NaCl, 136.9 mM; Na_2_HPO_4_, 10.14 mM; pH 7.2) and then resuspended in PBS to generate bacterial cell (BC) inocula (1x10^8^ CFU.mL^-1^). In subsequent sections, treatments derived from BC suspensions are denoted as (L), while those derived from CFE are denoted as (E).

### 2.3 *In vitro* seed germination and seedling vigor assays

#### 2.3.1 Seed germination assay

Common bean (*Phaseolus vulgaris* L. cv. ‘Gold Rush’; yellow bush bean type) seeds (W.H. Perron, QC, Canada) were surface sterilized by immersion in a dilution (30% v/v) of a commercial bleach solution (6% w/v NaOCl), corresponding to a final NaOCl concentration of approximately 1.8% w/v, for 10 min, followed by eight successive rinses with sterile distilled water (SDW). Sterilized seeds were then soaked in UBS for 1 h under light agitation at room temperature (RT). Following inoculation, seeds were placed in Petri dishes containing sterile paper towels moistened with SDW and incubated in the dark at RT. Each treatment consisted of six Petri plates containing 10 seeds each, for a total of 60 seeds per treatment. Seeds were considered germinated when radicle emergence reached a minimum length of 1 cm. Germination was recorded three days after initiation and expressed as the percentage of germinated seeds relative to the total number of seeds. Data were expressed as mean ± SD of six biological replicates per treatment.

#### 2.3.2 Seedling vigor assay

Seedling vigor was evaluated 7 days after germination by measuring total root length, defined as the sum of primary and lateral roots using ImageJ software (NIH, USA). Additional parameters included root fresh biomass, root dry biomass, specific root length (SRL), water content ratio on dry-biomass basis (WCR), and modified seedling vigor indices (SVI-I and SVI-II).

Root dry biomass was determined after oven-drying the young seedling roots at 40 °C for 72 hr. Measurements were expressed as mean ± SD of 15 biological replicates per treatment. The measurements were carried out as follows:

(1) Total root length =main root length (cm)+ lateral roots lengths (cm)

(2) $$WCR\;=\frac{root\;fresh\;biomass\;(mg)-root\;dry\;biomass\;(mg)}{root\;dry\;biomass\;(mg)}$$

(3) SRL =total root length (cm) / root dry biomass (mg)

(4) SVI−I =germination percentage (%)× total root length (cm)

(5) SVI−II =germination percentage (%)× root dry biomass (mg)

#### 2.3.3 Assessment of bacterial colonization in seedlings

At day 4, root sections from the BS-114 and BS-120 inoculated seedlings were collected and surface sterilized using the same protocol described above. The effectiveness of surface sterilization was verified by plating the final rinse water on LBA. The 1–1.5 cm root sections were macerated in 1 mL of SDW, and the resulting suspensions were serially diluted. A volume of 100 µL from each dilution was plated on LBA and incubated for 5–7 days. Phenotypically distinct colonies were selected and purified by repeated streaking to obtain single-colony isolates representing recovered endophytes.

Recovered isolates were first identified by 16S rRNA gene sequencing using the universal bacterial primers 27F (5′-AGAGTTTGATCCTGGCTCAG-3′) and 534R (5′-ATTACCGCGGCTGCTGG-3′) (Gagne‐Bourgue et al., 2013). Amplicons were sequenced by the Genome Quebec sequencing service (Montreal, QC, Canada), and the resulting sequences were analyzed using BLASTn for putative taxonomic identification. Following identification, isolates assigned to the genus *Bacillus* were selected for strain-level confirmation by housekeeping gene sequencing. The housekeeping genes *ilvD*, *pta*, *purH*, *pycA* were selected from the multilocus sequence typing (MLST) previously reported in the strain-characterization study (Kaddoura et al., 2026). The sequence obtained for each housekeeping gene was compared with the corresponding allele sequence identified from the WGS-derived MLST profile, and isolates were considered a match only when 100% sequence identity was observed across all selected housekeeping genes. The allelic profiles and primer sequences are provided in **Supplementary Tables 3**, **S4**.

### 2.4 Greenhouse experiments: validation under controlled conditions

The effects of bacterial inoculation on yellow bean health and productivity were evaluated using phenotypic and physiological parameters under controlled conditions. Greenhouse experiments were conducted in McGill University’s Greenhouse Facility (Macdonald Campus, QC, Canada).

Plants were grown in the commercial substrate AGRO MIX G6 (Fafard, QC, Canada). Treatments were assigned on a single greenhouse bench (4.4 m × 1.6 m) following a complete randomized design, with individual pots (8-inch diameter, 4 L volume) arranged at 20 cm spacing serving as the experimental units. Greenhouse environmental conditions were maintained at temperatures ranging from 18–26 °C, relative humidity 55–65%, and supplemental lighting provided by high-pressure sodium lamps at 250–350 μmol photons m^-2^ s^-1^, with a 16 h: 8 h (light: dark) photoperiod. Each pot was connected to two drip emitters supplying a total of 50 mL per pot per day for the first two weeks after transplanting, which was subsequently increased to 100 mL per pot per day for the remainder of the experiment. Common greenhouse pests were monitored using yellow sticky traps. Insecticidal soap and neem oil were applied judiciously and uniformly to prevent the spread of pests.

#### 2.4.1 Treatments and applications

##### 2.4.1.1 Trial 1: comparative assessment of bacterial treatments on plant growth and reproductive performance under greenhouse conditions

Treatments included BS-114 (20 mL UBS of BS-114 at 5% v/v), PPW-26 (20 mL UBS of PPW-26 at 5% v/v), LB (20 mL LB at 5% v/v) as a media control, CB (20 mL fermented algae-based commercial biostimulant control at 4% v/v) as a positive control, NPK (20 mL NPK 18-18–21 at 0.2% w/v) as a chemical control, and NC (20 mL of water) applied as a non-inoculated control.

Overnight bacterial cultures were adjusted based on OD_600_ = 0.8-0.9, corresponding to 1×10^8^ CFU.mL^-1^, prior to dilution for treatment application. Seeds were soaked for 1 hr at RT under light agitation in the respective bacterial treatment for BS-114 and PPW-26 or SDW for all other controls. Treatments were re-applied by soil drenching directly to the root zone at transplanting (week 1), trifoliate leaf development (week 2), flowering (week 5), and seed production (week 7), for a total of five treatment applications. The experiment consisted of six treatments arranged in a complete randomized design with five replicate pots per treatment.

##### 2.4.1.2 Trial 2: effects of bacterial formulations and NPK fertilization on plant performance under greenhouse conditions

Treatments included BS-114L (20 mL BC of BS-114 at 5% v/v), PPW-26L (20 mL BC of PPW-26 at 5% v/v), BS-114E (20 mL CFE of BS-114 at 5% v/v), PPW-26E (20 mL CFE of PPW-26 at 5% v/v), CS (20 mL total of BS-114L+PPW-26L at 5% v/v, 1:1 ratio), CS.NPK100 (20 mL CS followed by 20 mL NPK at 0.2% w/v), CS.NPK50 (20 mL CS followed by 10 mL NPK at 0.2% w/v), NPK (20 mL at 0.2% w/v), and NC.

Seeds were soaked for 1 hr at RT under light agitation in the corresponding bacterial cell treatment for BS-114L, PPW-26L, CS, CS.NPK100, and CS.NPK50, and in SDW for treatments that did not contain bacterial cells, including BS-114E, PPW-26E, and the remaining controls. Treatment applications followed the timeline outlined in 2.4.1.1, with NPK applications in combination treatments applied 1 hr after CS treatment. The experiment consisted of nine treatments arranged in a complete randomized design with eight replicate pots per treatment.

#### 2.4.2 Measured parameters and analytical methods

##### 2.4.2.1 Vegetative growth parameters

All vegetative growth indices were measured at the end of week 4, corresponding to the end of the vegetative stage prior to flowering. The parameters included leaf greenness, stem diameter, plant height, and total fresh mass. Leaf greenness was measured as SPAD units using a SPAD-502Plus chlorophyll meter (Konica, Minolta, Japan) to provide an estimate of leaf chlorophyll content. Measurements were taken from mature leaves at three canopy positions: the uppermost trifoliate, the middle trifoliate (third trifoliate from the top), and the lowest trifoliate located immediately above the unifoliate leaves. For each trifoliate, three SPAD readings were recorded per leaf, including both lateral and terminal leaflets. Mean SPAD values were calculated for each canopy position and subsequently averaged to obtain the mean SPAD value per plant. Stem diameter was measured using a digital caliper at the midpoint of the internode between the first and second nodes from the base of the plant. Plant height was measured using a measuring tape from the cotyledons to the shoot apical meristem. Total fresh mass per plant was measured at the final assessment using three biological replicates per treatment. At the time of measurement, any remaining pods were removed from the plant. Roots were washed to remove adhering substrate, and shoot and root fresh masses were summed to obtain the total fresh biomass per plant.

##### 2.4.2.2 Physiological parameters

Leaf gas-exchange parameters were measured using an LI-6800 portable photosynthesis system (LI-COR Biosciences, Lincoln, NE, USA). Measurements were conducted at week 4, at the end of the vegetative stage, between 8:00 and 10:00 AM under greenhouse conditions. Photosynthetic rate (A; μmol CO_2_ m^-2^ s^-1^), intercellular CO_2_ concentration (Ci; μmol mol^-1^), stomatal conductance (Gsw; mol H_2_O m^-2^ s^-2^), and transpiration rate (E; mol H_2_O m^-2^ s^-1^) were recorded. For each plant, the third fully expanded trifoliate leaf from the top, with uniform color and no visible damage, was selected for measurement. Two readings were recorded from the same leaf per plant and averaged prior to statistical analysis, with a total of eight plants measured per treatment. Chamber settings were maintained at a photosynthetic photon flux density of 1100 μmol photons m^-2^ s^-1^, CO_2_ concentration of 400 μmol mol^-1^, chamber temperature of 25°C, relative humidity of 60%, and flow rate of 600 μmol s^-1^.

##### 2.4.2.3 Reproductive parameters

Reproductive development was assessed by visually counting key reproductive structures per plant at defined developmental stages between the onset of budding (week 5) and the final harvest (week 9). Buds were counted during weeks 5-6, flowers at the end of week 6, immature pods (not fully developed) at week 7, and total pod number and fresh pod mass during the final harvest at week 9.

##### 2.4.2.4 Gene expression analysis by RT-qPCR

Total RNA was extracted from yellow bean leaves (20 mg) and roots (50 mg) at the end of the vegetative stage using the QIAGEN RNeasy ^®^ Mini Kit (QIAGEN, Hilden, Germany; Cat. No. 74104) following the manufacturer’s instructions. The extracted RNA was treated with DNase I (Bio-Rad Laboratories, Hercules, CA, USA; Cat. No. 100-42051) at 37°C for 30 min. RNA quality and concentration were assessed using a NanoDrop ND1000 spectrophotometer (Thermo Fisher Scientific, Waltham, MA, USA), and integrity was verified on 1% agarose gels. Reverse transcription was performed using the Agilent Affinity Script qPCR cDNA Synthesis Kit (Agilent Technologies, CA, USA; Cat. No. 60559).

All cDNA samples were diluted fivefold prior to amplification. qPCR reactions were carried out using PowerTrack™ SYBR Green Master Mix (Thermo Fisher Scientific, MA, USA; A46109) on an Mx3000P QPCR System (Agilent Technologies). Each reaction was performed in a 10 µL volume containing 0.5 µM of each primer and 1.5 µL diluted cDNA template. Thermocycling conditions followed a one-step PCR protocol described in **Supplementary Table 1**. Actin-11 (*Act11*) was used as the reference gene for normalization. Selected target genes associated with nitrogen metabolism and auxin- and cytokinin-related pathways were analyzed, including *NifH*, *AUX6*, *AUX*/*IAA*, *PVCK01*, *AIPT3*, and *LOG*/*LOG-like* genes. Primer sequences are provided in (**Supplementary Table 2**). The fluorescence threshold was set automatically by the instrument software, and amplification curves were analyzed accordingly. Relative gene expression levels were calculated using the 2^−ΔΔCT^ method from four technical replicates per treatment, derived from RNA pooled from six biological replicates, and expressed as mean fold change relative to the non-inoculated control.

### 2.5 Field experiments: validation under agronomic conditions

Bacterial treatment performance and effects on yellow bean productivity and soil microbial and chemical properties were investigated under field conditions in the summer of 2024.The experiment was conducted during the early growing season (May–July 2024) in Lavaltrie (QC, Canada), in clay loam soil. During the experimental period, mean daily air temperatures were 14.0–19°C in May, 17.6–23.0°C in June, and 21–26°C in July. Relative humidity ranged from 55% to 63% with an average dew point of 12.8°C.

Standard farmer management conditions were applied from planting to harvest, apart from the fertilization program, which was replaced by the experimental treatments. A randomized complete block design was implemented, consisting of four blocks per treatment, with a total of 60 (15×4) plants per treatment. Treatments were applied three times during the growing season by soil drenching at the root zone.

#### 2.5.1 Treatments and applications

Treatments included BS-114 (50 mL UBS at 5% v/v), BS-120 (50 mL UBS at 5% v/v), BS-114+BS-120 (50 mL total UBS of BS-114 and BS-120 at 5% v/v, 1:1 ratio), PPW-26 (50 mL UBS at 5% v/v), CS (50 mL total UBS of BS-114, BS-120, and PPW-26 at 5% v/v, 1:1:1 ratio), CB (fermented algae-based commercial biostimulant control at 0.6% v/v), chemical control NPK 18-18-21 (50 mL at 0.5% w/v), and non-inoculated control (NC).

#### 2.5.2 Sampling and data collection

Total yield from two harvests was recorded over the course of the experiment and reported as average pod fresh mass per treatment and average cumulative pod fresh mass per plant. Rhizospheric soil samples were collected from the root zone of five randomly selected plants per block. For each treatment, the samples were pooled to generate a single composite sample, which was subsequently divided into four replicates subsamples. All four subsamples were used for metagenomic analysis, while three were used for soil nutrient analysis.

##### 2.5.2.1 Soil bacterial community profiling

Bacterial community profiling was performed by targeting the V4 region of the 16S rRNA gene. Amplification was carried out with primers 515F (5′-GTGYCAGCMGCCGCGGTAA-3′) and 806R (5′-GGACTACNVGGGTWTCTAAT-3′), as previously described (Caporaso et al., 2011). Libraries were sequenced on an Illumina MiSeq platform using V3 chemistry (2 × 300 bp) paired-end reads at the McGill Genome Centre (Montreal, QC, Canada). Reagent-only controls were processed in parallel and confirmed that contamination was below the detection threshold. Raw reads were processed using DADA2 (v1.20.0). Adapter and primer sequences were trimmed using Cutadapt (v2.10), after which reads shorter than 180 bp or lacking primer sequences were excluded. Remaining reads were quality-filtered with a maximum allowance of two expected errors per read, truncated at a Phred score ≤2, and trimmed to 200 bp for both forward and reverse reads to reduce low-quality terminal bases. PhiX-derived reads were also removed. Amplicon sequence variants (ASVs) were inferred by merging overlapping reads with a minimum overlap of 20 bp. Chimeric sequences were removed using DADA2’s “removeBimeraDenovo()” function. Taxonomic assignment was then performed using a naïve Bayesian classifier trained on the SILVA (v138) reference database.

##### 2.5.2.2 Soil nutrient analysis

Soil samples were analyzed for physicochemical properties by A&L Canada Laboratories Inc. (ON, Canada) using standardized protocols (Page, 1982; Tran and Simard, 1993; U.S. Environmental Protection Agency, 1993). Measured parameters included soil organic matter, pH, Mehlich III-extractable macro- and micronutrients, inorganic nitrogen forms (NH_4_^+^, NO_3_^-^, NO_2_^-^), total Kjeldahl nitrogen, total carbon and nitrogen, and the carbon-to-nitrogen (C:N) ratio. Mean values were calculated from four replicates per treatment.

### 2.6 Statistical analyses

Data processing and analyses were performed using Microsoft Excel and GraphPad Prism (v10.6.1; GraphPad Software, CA, USA). Seed and greenhouse 1 treatments were evaluated using one-way analysis of variance (ANOVA), followed by Dunnett’s *post-hoc* test with NC. Greenhouse 2 treatments were compared using one-way ANOVA followed by Tukey’s *post-hoc* test for pairwise comparisons. Field treatments were compared using one-way ANOVA based on block-level averages followed by Tukey’s *post-hoc* test. For soil bacterial community profiling, alpha diversity indices (Shannon diversity and observed ASVs) were calculated, and differences among treatments were assessed using one-way ANOVA followed by Tukey’s *post-hoc* test. All results are presented as mean ± standard deviation, with significance set at p< 0.05.

## 3 Results

### 3.1 Seed germination, seedling vigor, and bacterial colonization

**Figure 1:**
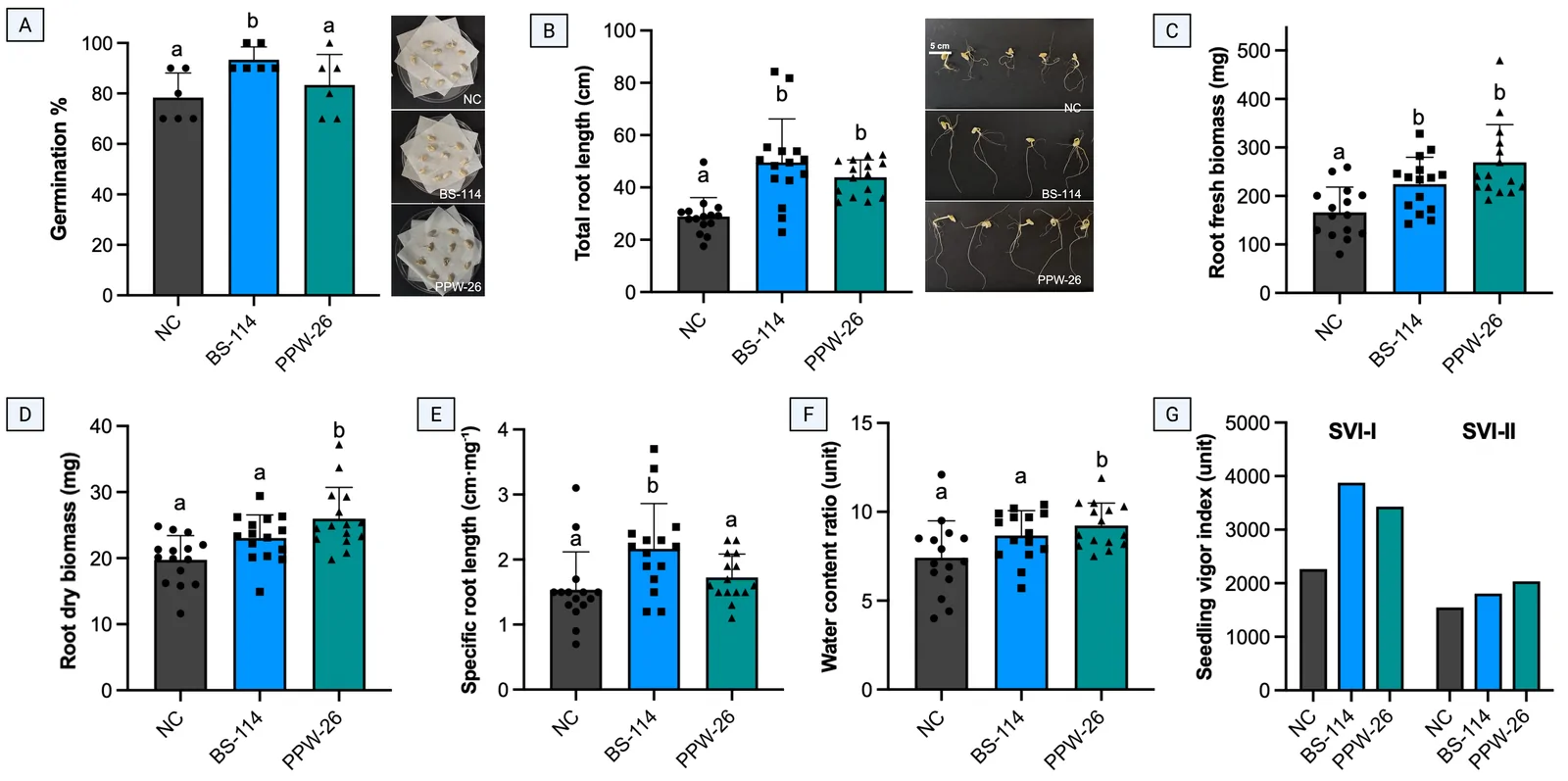
Seed germination at day 3 and seedling parameters at day 7 of treated yellow bean seeds. NC, non-inoculated control; BS-114, *B. subtilis* strain 114 (UBS); PPW-26, *P. wadenswilerensis* strain 26 (UBS). **(A)** Percentage germination **(B)** Total root length **(C)** Root fresh biomass **(D)** Root dry biomass **(E)** Specific root length **(F)** Water content ratio **(G)** Seedling vigor index -I and -II. Values represent mean ± SD (n= 6 for seed germination, n= 15 for seedling vigor). Different letters indicate significant difference relative to the control according to one-way ANOVA followed by Dunnett’s *post-hoc* test (P < 0.05).

Throughout the manuscript, all reported percentage changes are presented relative to NC unless otherwise specified. In addition, the terms “numerical increase” and “numerically higher” indicate increases that did not meet the threshold for statistical significance (P < 0.05). At day 3, seed germination was significantly higher under BS-114 (93.3%; +19.1% relative to NC), whereas PPW-26 (83.3%; +6.4%) showed a smaller numerical increase (**Figure 1A**). Among the seedling vigor parameters, significant increases under both bacterial treatments were observed in total root length, with BS-114 (+71.3%) and PPW-26 (+51.7%), and root fresh biomass, with BS-114 (+34.9%) and PPW-26 (+61.7%) (**Figures 1B, C**). Root dry biomass increased significantly only under PPW-26 (+31.6%) and numerically under BS-114 (+16.7%) (**Figure 1D**). Specific root length showed a significant increase under BS-114 (+40.1%) and PPW-26 (+12.2%), while water content ratio was significantly higher under PPW-26 (+24.5%) and numerically higher under BS-114 (+16.9%) (**Figures 1E, F**). Finally, both seedling vigor indices were higher under bacterial treatments, with SVI-I highest under BS-114 (+71.3%) and SVI-II highest under PPW-26 (+31.6%), respectively (**Figure 1G**).

*Bacillus* isolates recovered from the roots of BS-114-inoculated seedlings showed 100% sequence identity to the expected BS-114 allele sequences at all selected housekeeping loci: *ilvD* allele 48, *pta* allele 55, *purH* allele 49, and *pycA* allele 34. Several other candidate isolates, including those recovered from BS-120-inoculated seedlings, matched only a subset of the BS-114 allele sequences and were therefore excluded as BS-114. Collectively, the complete allele matches corresponded to the reported MLST allelic profile of BS-114, strongly supporting the recovery of BS-114 from internal bean root tissues. In contrast, isolates recovered from BS-120-inoculated seedlings did not match the expected BS-120 allele sequences across all selected housekeeping loci; therefore, colonization by the inoculated BS-120 strain could not be confirmed.

### 3.2 Comparative assessment of bacterial treatments on plant growth and reproductive performance under greenhouse conditions

#### 3.2.1 Vegetative growth parameters

**Figure 2:**
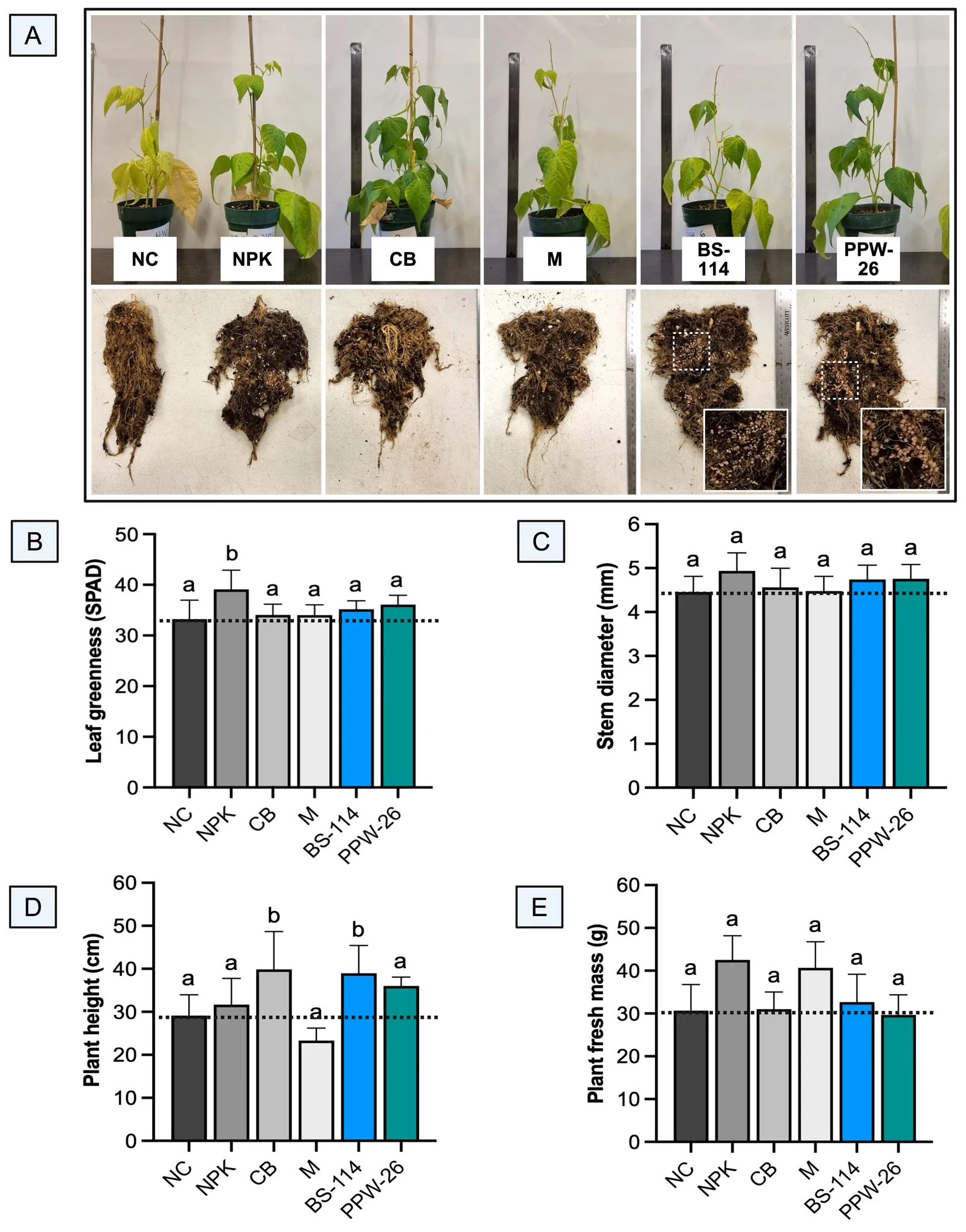
Vegetative growth parameters at week 4 and representative final plant phenotypes at week 9 of treated yellow bean plants. NC, non-inoculated control; NPK, chemical control; CB, commercial biostimulant control; M, media control; BS-114, *B. subtilis* strain 114 (UBS); PPW-26, *P. wadenswilerensis* strain 26 (UBS). **(A)** Representative images of plants and root phenotypes **(B)** Leaf greenness **(C)** Stem diameter **(D)** Plant height **(E)** Total fresh mass. Values represent mean ± SD (n= 5). Different letters indicate significant difference relative to the control according to one-way ANOVA followed by Dunnett’s *post-hoc* test (P < 0.05).

Vegetative growth parameters revealed varying physiological and phenotypic responses. Representative images at week 9 showed treatment-level differences in plant phenotype relative to NC (**Figure 2A**), with increased visible nodulation under both BS-114 and PPW-26. Leaf greenness differed among treatments, with NPK exhibiting a significant increase (+17.6%), followed by numerical increases under PPW-26 (+8.7%), BS-114 (+5.8%), CB (+2.5%), and M (+2.3%) (**Figure 2B**). Stem diameter showed no significant differences among treatments; however, slight increases were observed for NPK (+10.8%), PPW-26 (+6.7%), BS-114 (+6.3%), CB(+2.2%), and M (+0.4%) (**Figure 2C**). Plant height varied at the end of the vegetative stage, with CB and BS-114 showing significant increases of +37.0% and +33.9%, respectively, followed by numerical increases under PPW-26 (+23.8%), and NPK (+8.7%) (**Figure 2D**). Lastly, total fresh weight per plant showed the highest numerical increases under NPK (+38.6%) and M (+32.6%), while modest numerical increases were observed under BS-114 (+6.5%) and CB (+1.1%) (**Figure 2E**).

#### 3.2.2 Reproductive parameters

**Figure 3:**
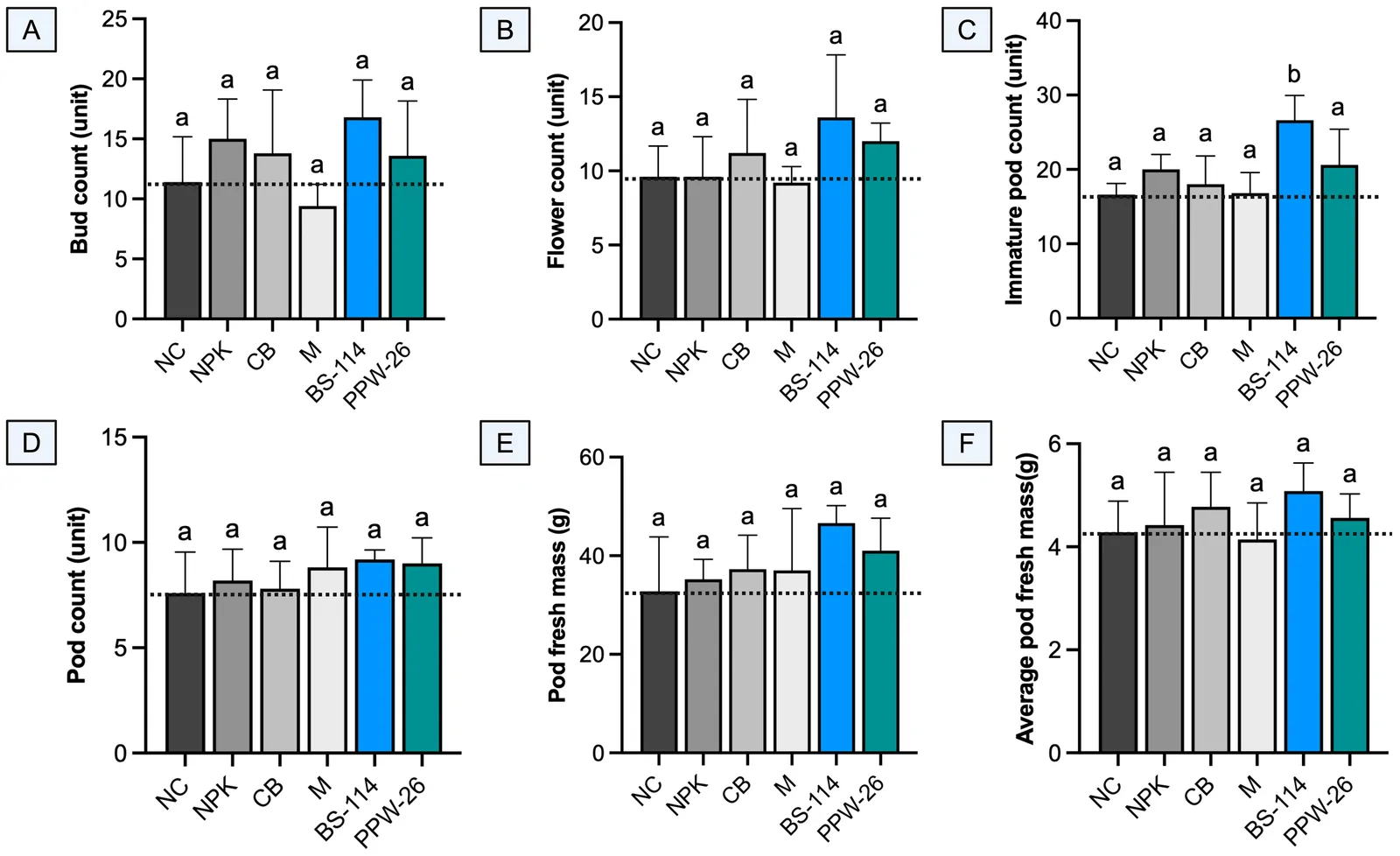
Reproductive parameters of treated yellow bean plants. NC, non-inoculated control; NPK, chemical control; CB, commercial biostimulant control; M, LB media control; BS-114, *B. subtilis* strain 114 (UBS); PPW-26, *P. wadenswilerensis* strain 26 (UBS). **(A)** Bud count at week 6 **(B)** Flower count at week 6 **(C)** Immature pod count at week 7 **(D)** Cumulative pod count at weeks 8-9 **(E)** Cumulative pod fresh mass at weeks 8-9 **(F)** Average pod fresh mass at weeks 8-9. Values represent mean ± SD (n= 5). Different letters indicate significant difference relative to the control according to one-way ANOVA followed by Dunnett’s *post-hoc* test (P < 0.05).

Yield-related traits also varied among treatments. Bud counts were numerically higher under BS-114 (+47.4%), followed by NPK (+31.6%), CB (+21.1%), and PPW-26 (+19.3%) (**Figure 3A**). Similarly, flower counts showed numerical increases under BS-114 (+41.7%), PPW-26 (+25.0%) and CB (+16.7%) (**Figure 3B**). Early assessment of pod development at week 6, revealed differences in pod onset among treatments, with the highest immature pod counts under BS-114 (1.6 pods; +166.7%), followed by PPW-26 (1.0 pods; +66.7%), and CB (0.8 pods; +33.3%), whereas NC and NPK both exhibited 0.6 pods, and M recorded no pods at this stage (**Supplementary Figure 1**). At week 7, immature pod counts increased significantly under BS-114 (+60.2%) and numerically under PPW-26 (+24.1%), NPK (+20.5%), CB (+8.4%) and M (+1.2%) (**Figure 3C**). Cumulative mature pod counts were numerically higher under BS-114 (+21.1%), PPW-26 (+18.4%), M (+15.8%), NPK (+7.9%) and CB (+2.6%) (**Figure 3D**), while cumulative mature pod fresh mass was numerically higher under BS-114 (+42.1%), followed by PPW-26 (+25.0%), CB (+13.4%), M (+12.8%), and NPK (+7.3%) (**Figure 3E**). Lastly, only three treatments showed improvements in average pod fresh mass, with the highest increase under BS-114 (+17.4%), CB (+10.5%), and PPW-26 (+5.6%) (**Figure 3F**).

### 3.3 Effects of bacterial formulations and NPK fertilization on plant performance under greenhouse conditions

#### 3.3.1 Vegetative growth and physiological parameters

**Figure 4:**
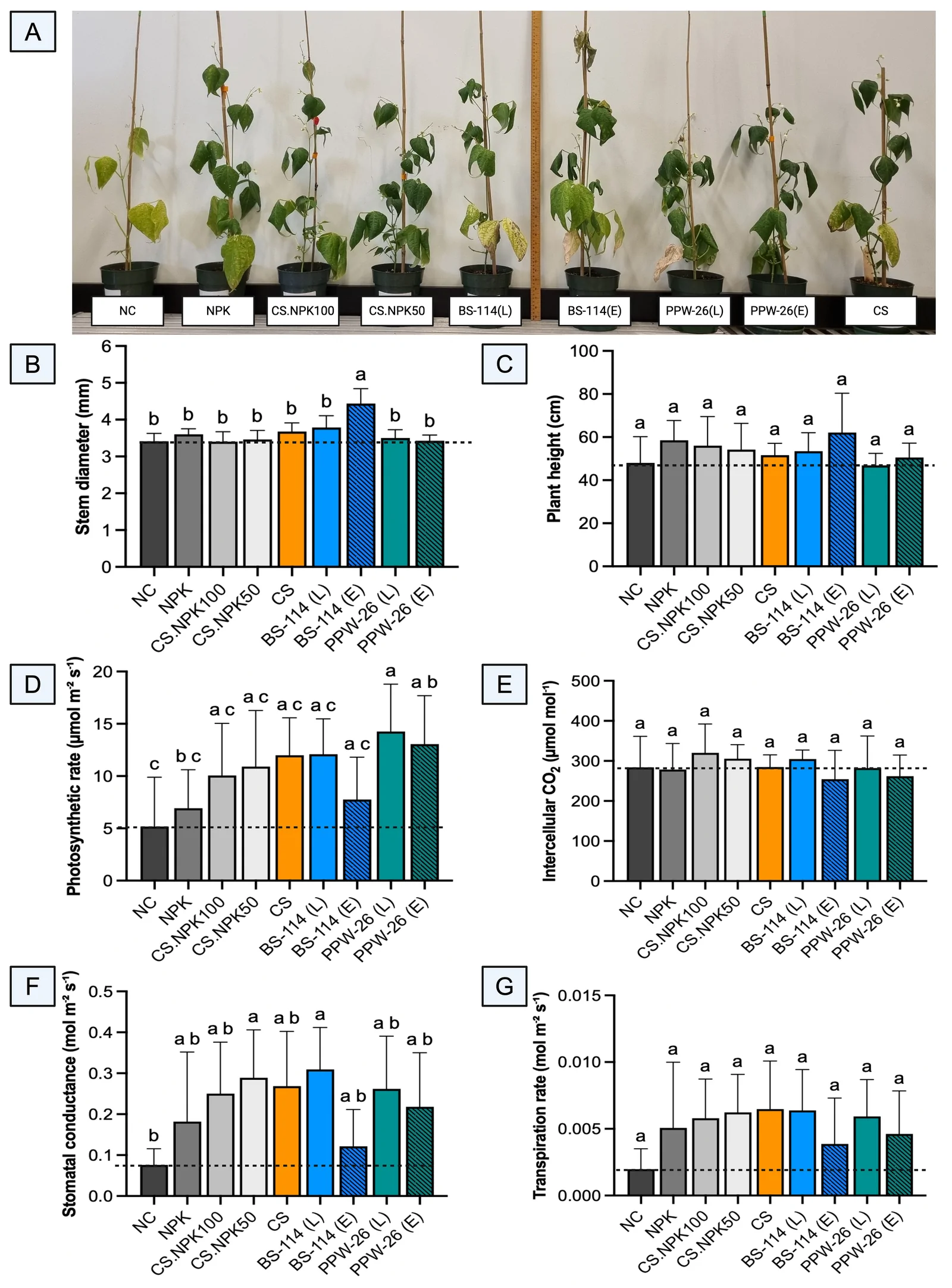
Vegetative growth (week 4) and physiological (week 5) parameters of treated yellow bean plants. NC, non-inoculated control; NPK, chemical control; CS.NPK100, bacterial consortia + chemical control 100% recommended dose; CS.NPK50, bacterial consortia + chemical control 50% recommended dose; CS, bacterial consortia BS-114L+PPW-26L; BS-114L, *Bacillus subtilis* strain 114 (BC), BS-114E; *Bacillus subtilis* strain 114 (CFE); PPW-26L, *Pseudomonas wadenswilerensis* strain 26 (BC); PPW-26E, *Pseudomonas wadenswilerensis* strain 26 (CFE). **(A)** Representative phenotypes of treatments **(B)** Stem diameter **(C)** Plant height **(D)** Photosynthetic rate **(E)** Intercellular CO_2_ **(F)** Stomatal conductance **(G)** Transpiration rate. Values represent mean ± SD (n= 8) according to one-way ANOVA followed by *post-hoc* Tukey’s test with different letters indicative of significant differences (P < 0.05) between treatments.

Among the measured vegetative growth parameters, stem thickness increased significantly under BS-114E (+29.0%) while small numerical increases were observed under CS (+9.7%), BS-114L (+9.2%), NPK (+6.3%), PPW-26L (+5.0%), CS.NPK50 (+2.5%), and PPW-26E (+1.7%) (**Figure 4A**). Plant height did not differ significantly across treatments; however, numerical increases were observed, under BS-114E (+24.8%) and NPK (+22.5%), CS.NPK100 (+15.8%), CS.NPK50 (+8.4%), BS-114L (+5.0%), CS (+4.9%), and PPW-26E (+3.1%) (**Figure 4C**).

Physiological parameters showed variable responses across treatments. Photosynthetic rate increased significantly under PPW-26L (+175.8%), and numerically under PPW-26E (+152.8%), BS-114L (+133.9%), CS (+132.1%), CS.NPK50 (+111.2%), CS.NPK100 (+94.6%), BS-114E (+50.0%), and NPK (+34.2%) (**Figure 4D**). Intercellular CO_2_ levels showed numerical improvements under CS.NPK100 (+12.8%), BS-114L (+7.4%), CS.NPK50 (+7.6%), and CS (+0.2%) (**Figure 4E**). Stomatal conductance varied, with the highest significant increases under BS-114L (+306.9%) and CS.NPK50 (+279.9%), followed by numerical increases under CS (+253.1%), PPW-26L (+244.6%), CS.NPK100 (+228.9%), PPW-26E (+187.1%), NPK (+139.2%), and BS-114E (+59.8%) (**Figure 4F**). Transpiration rate showed numerical increases under CS (+226.2%), BS-114L (+220.8%), CS.NPK50 (+214.2%), PPW-26L (+199.1%), CS.NPK100 (+191.6%), NPK (+155.4%), and PPW-26E (+132.0%) (**Figure 4G**).

#### 3.3.2 Reproductive parameters

**Figure 5:**
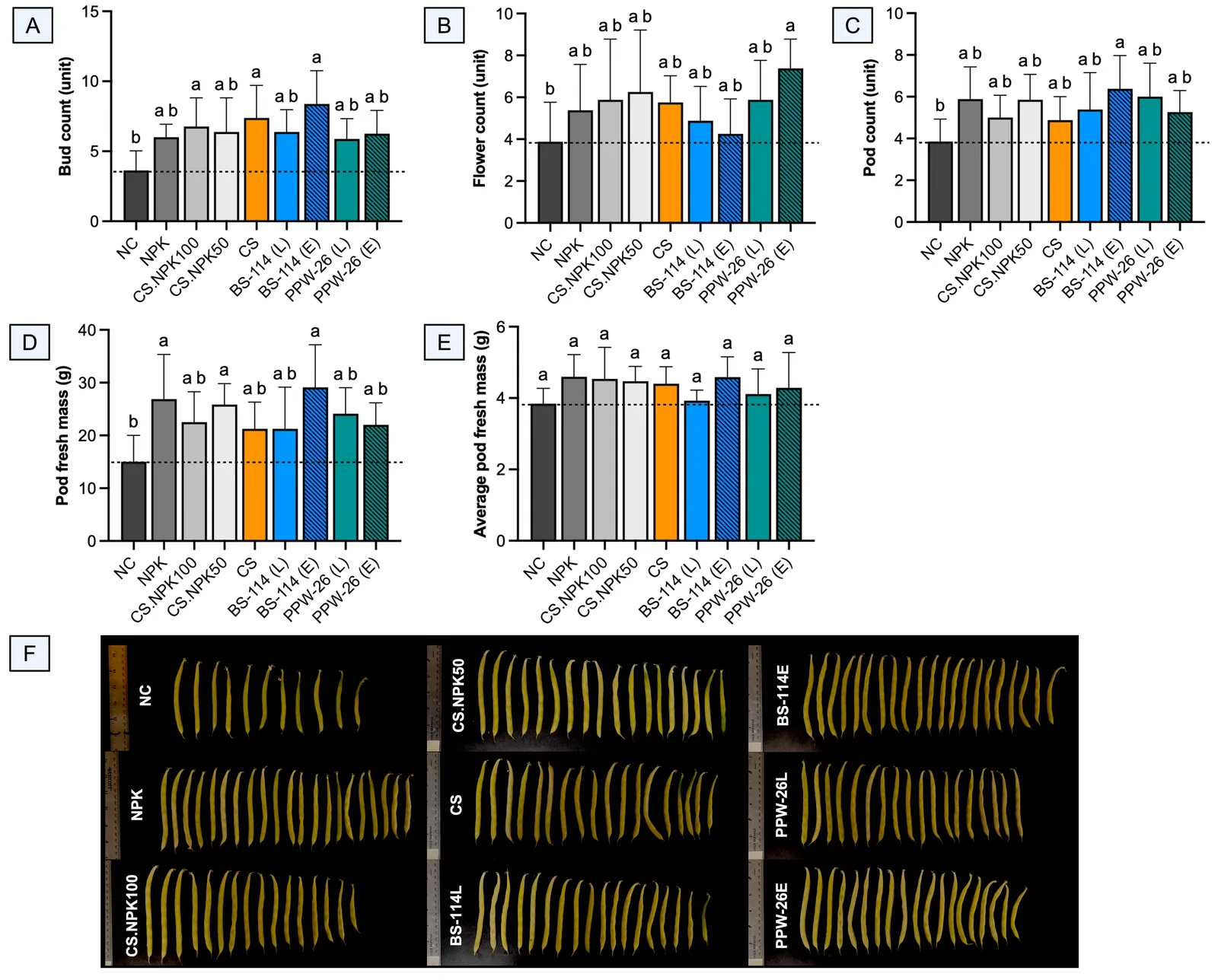
Reproductive parameters of treated yellow bean plants. NC, non-inoculated control; NPK, chemical control; CS.NPK100 (bacterial consortia + chemical control 100% recommended dose); CS.NPK50 (bacterial consortia + chemical control 50% recommended dose); CS, bacterial consortia BS-114L+PPW-26L; BS-114L, *B. subtilis* strain 114 (BC), BS-114E; *B. subtilis* strain 114 (CFE); PPW-26L, *P. wadenswilerensis* strain 26 (BC); PPW-26E, *P. wadenswilerensis* strain 26 (CFE). **(A)** Bud count at week 6 **(B)** Flower count at week 6 **(C)** Cumulative pod count at weeks 8-9 **(D)** Cumulative pod fresh mass at weeks 8-9 **(E)** Average pod fresh mass at weeks 8-9 **(F)** Representative image of treatment pods. Values represent mean ± SD (n= 8) according to one-way ANOVA followed by *post-hoc* Tukey’s test with different letters indicative of significant differences (P < 0.05) between treatments.

Reproductive parameters also varied across treatments and developmental stages. Bud count was significantly higher under BS-114E (+131.0%), followed by CS (+103.4%), and CS.NPK100 (+6.2%), with numerical increases under BS-114L and CS.NPK50 (+75.9%), PPW-26E (+72.4%), NPK (+65.5%), and PPW-26L (+62.1%) (**Figure 5A**). Flower counts increased significantly under PPW-26E (+90.3%), and numerically under CS.NPK50 (+61.3%), PPW-26L and CS.NPK100 (+51.6%), CS (+48.4%), NPK (+38.7%), BS-114L (+25.8%), and BS-114E (+9.7%) (**Figure 5B**). Cumulative pod count was significantly higher under BS-114E (+65.3%), with numerical increases under PPW-26L (+55.6%), NPK and CS.NPK50 (+52.7%), BS-114L (+39.4%), PPW-26E (+36.1%), CS.NPK100 (+29.6%), and CS (+26.4%) (**Figure 5C**). Cumulative pod fresh mass was significantly higher under BS-114E (+94.2%), NPK(+79.3%), and CS.NPK50 (+72.4%), with numerical increases under PPW-26L (+60.8%), CS.NPK100 (+50.0%), PPW-26E (+46.7%), and BS-114L and CS (+41.7%) (**Figure 5D**). Lastly, average pod fresh mass showed numerical increases, with the highest under NPK (+52.7%), followed by BS-114E (+19.1%), CS.NPK100 (+17.7%), CS.NPK50 (+16.0%), CS (+14.1%), PPW-26E (+11.5%), PPW-26L (+6.9%), and BS-114L (+1.7%) (**Figures 5E, F**).

#### 3.3.3 Gene expression analysis by RT-qPCR

**Figure 6:**
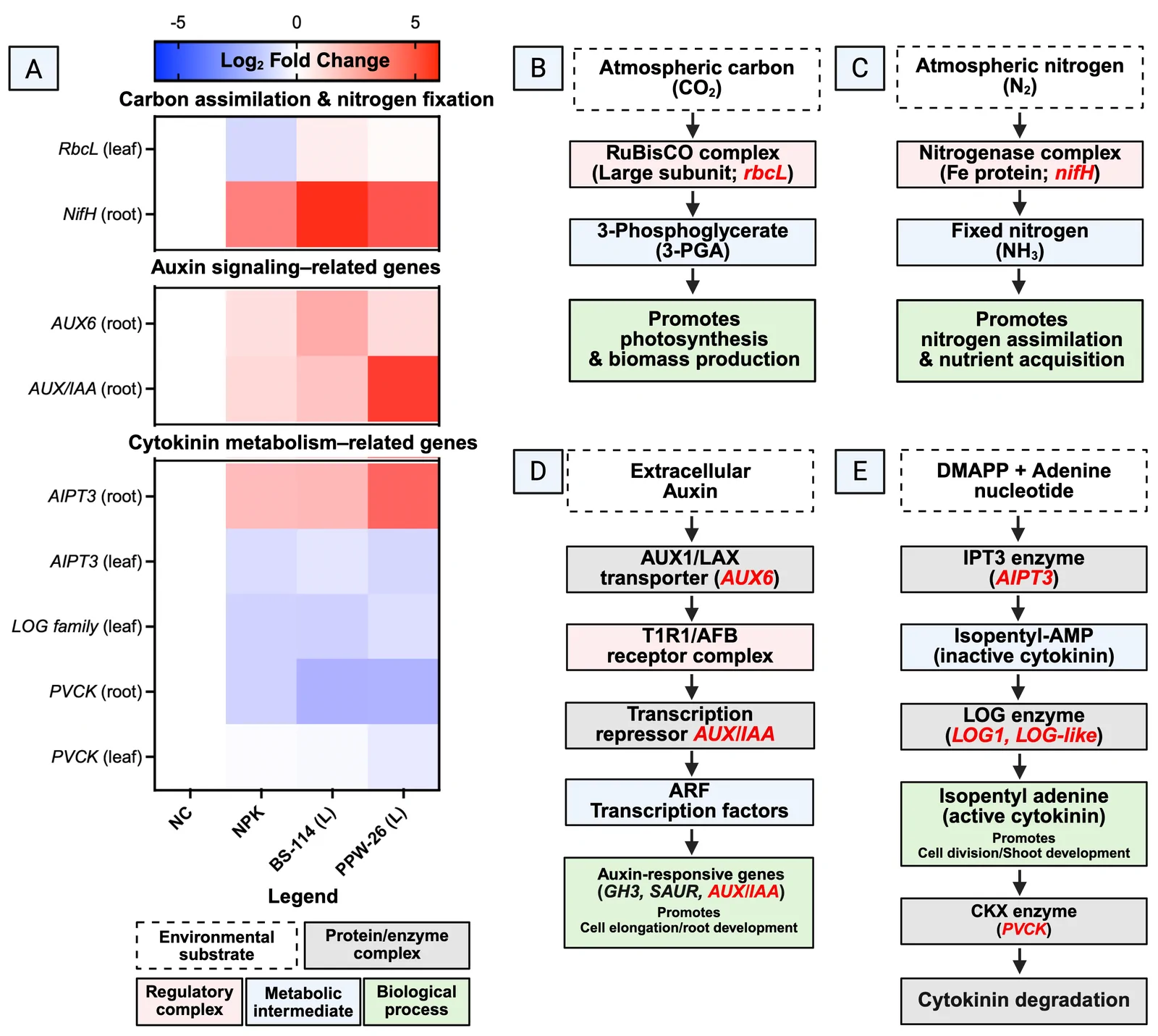
**(A)** Relative gene expression and simplified pathways of selected genes associated with **(B)** carbon assimilation, **(C)** nitrogen fixation, **(D)** auxin signaling, and **(E)** cytokinin metabolism and signaling. NC, non-inoculated control; NPK, chemical control; BS-114L, *B. subtilis* strain 114 (BC); and PPW-26L, *P. wadenswilerensis* strain 26 (BC). *rbcL*, ribulose-1, 5-bisphosphate carboxylase/oxygenase large subunit; *nifH*, nitrogenase iron protein; *AUX6*, auxin transporter-like protein 2; *AUX/IAA*, auxin-responsive protein; *AIPT3*, adenylate isopentenyltransferase 3; *LOG1/LOG1-like*, cytokinin riboside 5′-monophosphate phosphoribohydrolase (LOG family); and *PVCK*, cytokinin oxidase. Expression values are presented as log_2_ fold change relative to NC. Color scale represents downregulation (blue) to upregulation (red).

Expression of growth- and development-related genes was evaluated in plants treated with live bacterial cells and compared with NC (**Figure 6A**). Expression was assessed in leaves and roots at the end of week four, corresponding to the vegetative stage prior to the onset of the reproductive phase. The selected genes represent key physiological pathways including carbon assimilation, nitrogen fixation, auxin signaling, and cytokinin metabolism (**Figures 6B–E**). A total of nine genes were selected for qPCR analysis and *Act11* was used as the reference gene.

The carbon fixation-related gene ribulose-1, 5-bisphosphate carboxylase/oxygenase large subunit (*RbcL*) expression in leaves showed minor variation, with slight increases in BS-114L (+0.50 log_2_FC; 1.42×FC) and PPW-26L (+0.13 log_2_FC; 1.10×FC), while NPK showed reduced expression (-1.22 log_2_FC; 0.43×FC). Within the nitrogen fixation pathway, root expression of the nitrogenase reductase gene (*NifH*) showed strong upregulation, with the highest expression observed in BS-114L (+5.62 log_2_FC; 49.31×FC), followed by PPW-26L (+4.63 log_2_FC; 24.77×FC) and NPK (+3.58 log_2_FC; 11.96×FC).

Within the auxin signaling pathway, root expression of the auxin influx transporter (*AUX6*) was highest in BS-114L (+2.42 log_2_FC; 5.36×FC), followed by PPW-26L (+1.05 log_2_FC; 2.07×FC) and NPK (+0.96 log_2_FC; 1.95×FC). Root expression of the auxin-responsive transcriptional repressor (*AUX/IAA*) was highest in PPW-26L (+5.24 log_2_FC; 37.89×FC), followed by BS-114L (+1.76 log_2_FC; 3.39×FC), and NPK (+1.14 log_2_FC; 2.21×FC).

Within the cytokinin signaling pathway, root expression of adenylate isopentenyltransferase 3 (*AIPT3*) was highest in PPW-26L (+4.25 log_2_FC; 18.99×FC), followed by BS-114L (+2.06 log_2_FC; 4.17×FC), and NPK (+1.97 log_2_FC; 3.92×FC). In leaves, *AIPT3* expression decreased slightly across treatments, BS-114L (-0.82 log_2_FC; 0.57×FC), PPW-26L (-1.21 log_2_FC; 0.43×FC), and NPK (-1.09 log_2_FC; 0.47×FC). Leaf expression of the cytokinin phosphoribohydrolase (*LOG1/LOG-like*) showed small reductions relative to NC, with PPW-26L (-0.99 log_2_FC; 0.51×FC), NPK (-1.38 log_2_FC; 0.39×FC), and BS-114L (-1.42 log_2_FC; 0.37×FC). Root expression of cytokinin oxidase/dehydrogenase (*PVCK01*) was downregulated across all treatments, with NPK (-1.37 log_2_FC; 0.39×FC), PPW-26L (-2.27 log_2_FC; 0.21×FC), and BS-114L (-2.29 log_2_FC; 0.20×FC). A similar downregulation was observed in leaves, with NPK (-0.14 log_2_FC; 0.91×FC), BS-114L (-0.22 log_2_FC; 0.86×FC), and PPW-26 (-0.68 log_2_FC; 0.63×FC).

### 3.4 Field experiments: validation under agronomic conditions

#### 3.4.1 Yield parameters

**Figure 7:**
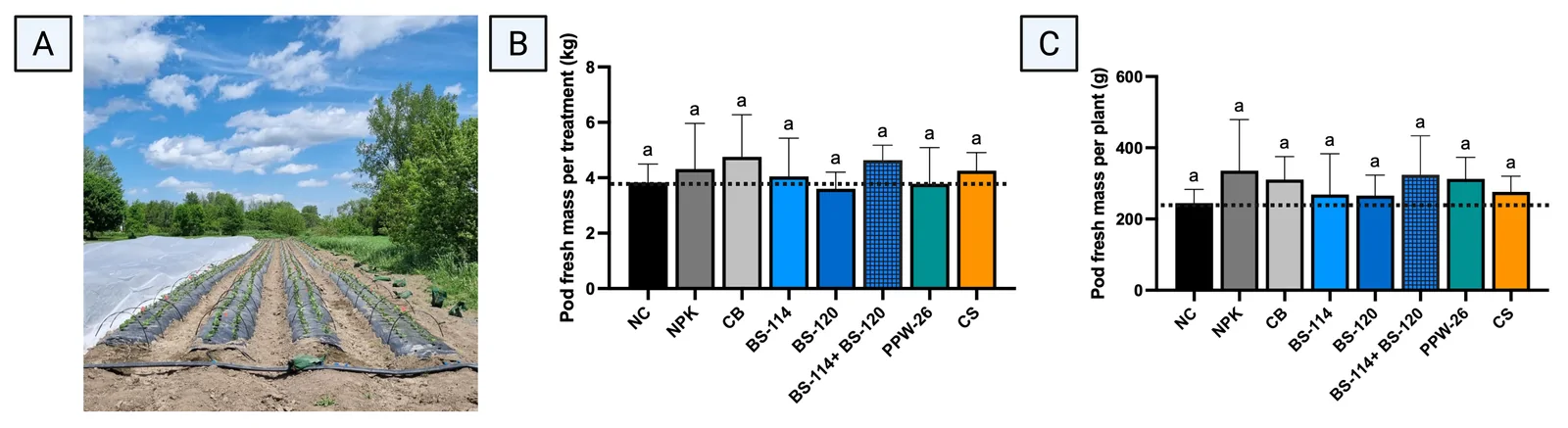
Field performance of yellow bean plants. NC, non-inoculated control; NPK, chemical control; CB, fermented algae-based commercial biostimulant control; BS-114, *B. subtilis* strain 114 (UBS); BS-120, *B. subtilis strain* 120 (UBS); BS-114+BS-120, *B. subtilis strain* 114 (UBS) + strain 120 (UBS); PPW-26, *P. wadenswilerensis* strain 26 (UBS); CS, BS-114+BS-120+PPW-26. **(A)** Representative field trial image **(B)** Pod fresh mass per treatment **(C)** Pod fresh mass per plant. Values represent mean ± SD (n= 4) according to one-way ANOVA followed by *post-hoc* Tukey’s test, with different letters indicative of significant differences (P < 0.05) between treatments.

Field trials conducted during the summer of 2024 showed variation in yield among treatments relative to the non-inoculated control (NC) (**Figures 7B, C**). For average pod fresh mass per treatment, the highest values were observed under CB and BS-114 + BS-120, followed by NPK and CS. For average pod fresh mass per plant, NPK showed the highest increase (+37.76%; 0.338 kg), followed by BS-114+BS-120 (+32.65%; 0.325 kg), CB and PPW-26 (27.55%; 0.313 kg), CS (13.27%; 0.278 kg), whereas BS-114 and BS-120 showed the lowest increases (+9.69%; 0.268 kg). However, no statistically significant differences were observed among treatments.

#### 3.4.2 Soil bacterial community profiling

**Figure 8:**
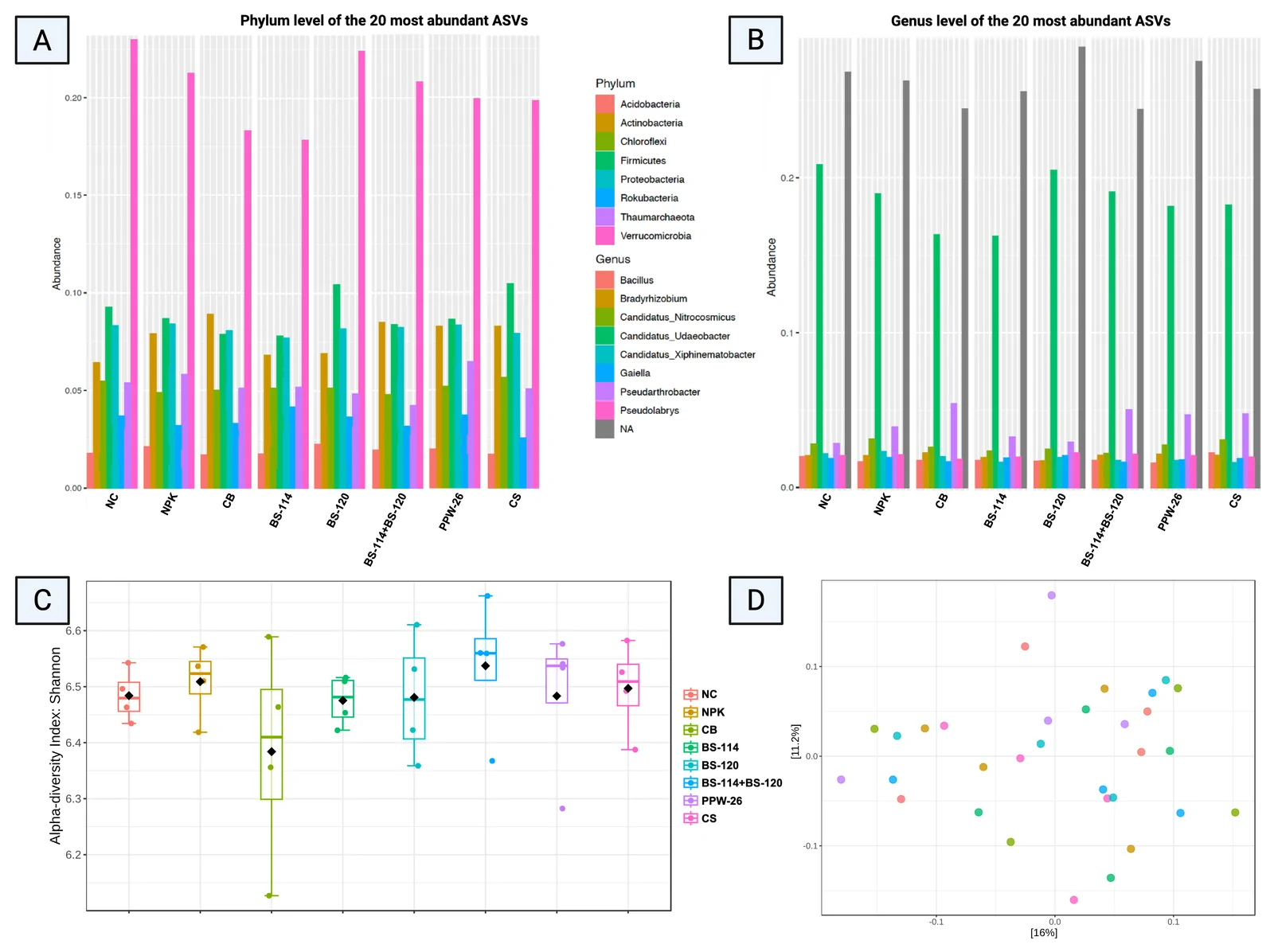
Bacterial community composition and diversity across treatment groups. **(A)** Relative abundance of bacterial taxa at the phylum level **(B)** Relative abundance of bacterial taxa at the genus level **(C)** Alpha diversity assessed using the Shannon index **(D)** Beta diversity based on principal coordinate analysis (PCoA).

Following quality filtering, denoising, and chimera removal, sequencing depth ranged from 56, 832 to 94, 560 reads per sample across treatments. Observed ASV richness ranged from 1, 454 to 2, 251 ASVs per sample. Alpha diversity based on the Shannon index showed comparable diversity across treatments, ranging from 6.84 (PPW-26) to 6.97 (CB) (**Figure 8C**). Bacterial community profiling revealed the distribution of amplicon sequence variants across taxonomic levels as follows: 2% at the kingdom level, 4% at the phylum level, 12% at the class level, 13% at the order level, 34% at the family level, 32% at the genus level, and 2% at the species level. At the phylum level, microbial communities were dominated by *Verrucomicrobia*, followed by *Actinobacteria*, *Proteobacteria*, and *Firmicutes* across all samples (**Figure 8A**). Additional phyla detected at lower relative abundances included *Chloroflexi*, *Rokubacteria*, and *Thaumarchaeota*. Minor variations in relative abundance among treatments were observed across these dominant phyla. At the genus level, microbial community composition differed among treatments relative to NC (**Figure 8B**). Unidentified genera collectively accounted for less than 0.3 relative abundance across all samples. The archaeal genus *Candidatus Nitrocosmicus* was more abundant in NPK and bacterial treatments. Notably, *Pseudarthrobacter* was markedly abundant in the BS-114+BS-120 treatment compared with other treatments. The genus *Bradyrhizobium* was detected across all treatments with slight variations in relative abundance, while *Bacillus* was also present in all samples. Overall, treatment-related differences in microbial community composition were observed at both the phylum and genus levels, although these did not result in clear separation among treatments in beta diversity analysis (**Figure 8D**).

#### 3.4.3 Soil nutrient analysis

Soil nutrient analysis revealed significant differences in several soil fertility parameters among treatments (**Table 1**). C:N ratio, nitrite, and pH did not differ significantly among treatments. Several tested parameters, including organic matter, magnesium, calcium, sodium, and sulfur, showed significant differences among treatments; however, these did not follow a clear trend across bacterial treatments. Among the measured nitrogen forms, significantly differences were observed for nitrate-N, ranging from 12.33 ppm (PPW-26) to 28.00 ppm (CB), ammonia ranging from 3.33 ppm (BS-114+BS-120) to 13.33 ppm (PPW-26), and total Kjeldahl nitrogen ranging from 0.22% (BS-114 and PPW-26) to 0.27% (CB). Similarly, bicarbonate-extractable phosphorus ranged from 63.33 ppm (PPW-26) to 86.00 ppm (NPK), while potassium ranged from 91.00 ppm (BS-114+BS-120) to 138.67 ppm (CB).

**Table 1:** Summary of rhizospheric soil nutrient analysis.

Treatment	OM (%)	C:N	NO_2-_ (ppm)	NO_3-_-N (ppm)	NH_3_ (ppm)	TKN (%)	P-HCO_3-_ (ppm)	K^+^ (ppm)	Mg^2+^ (ppm)	Ca^2+^ (ppm)	Na^+^ (ppm)	S (ppm)	pH
NC	4.40 ± 0.10^ac^	10.47 ± 0.51^a^	1.00 ± 0.00^a^	16.67 ± 1.53^bc^	4.33 ± 2.31^ab^	0.24 ± 0.02^ab^	65.33 ± 2.52^c^	111.67 ± 2.52^cd^	266.00 ± 7.21^ab^	2006.67 ± 51.32^ab^	26.33 ± 1.53^c^	9.33 ± 0.58^b^	7.07 ± 0.06^a^
NPK	4.67 ± 0.06^a^	10.50 ± 0.20^a^	1.33 ± 0.58^a^	17.33 ± 1.53^bc^	5.00 ± 2.65^ab^	0.24 ± 0.01^ab^	86.00 ± 1.73^a^	125.33 ± 1.15^ab^	236.67 ± 3.21^d^	2100.00 ± 26.46^a^	27.33 ± 0.58^bc^	12.33 ± 0.58^ab^	7.20 ± 0.10^a^
CB	4.57 ± 0.12^ab^	10.43 ± 0.25^a^	1.67 ± 0.58^a^	28.00 ± 1.00^a^	6.33 ± 2.89^ab^	0.27 ± 0.02^a^	85.00 ± 3.46^a^	138.67 ± 7.23^a^	247.67 ± 4.04^bd^	2023.33 ± 32.15^ab^	27.33 ± 1.15^bc^	14.33 ± 1.15^a^	6.97 ± 0.12^a^
BS-114	4.23 ± 0.06^c^	11.07 ± 0.38^a^	2.00 ± 1.73^a^	18.00 ± 1.00^bc^	3.67 ± 0.58^ab^	0.22 ± 0.01^b^	68.33 ± 1.15^c^	99.67 ± 4.51^de^	272.33 ± 9.50^a^	2106.67 ± 80.21^a^	28.00 ± 1.73^ac^	12.33 ± 2.31^ab^	7.20 ± 0.10^a^
BS-120	4.50 ± 0.10^ac^	10.37 ± 0.74^a^	1.00 ± 0.00^a^	19.67 ± 4.51^b^	4.33 ± 1.53^ab^	0.24 ± 0.01^ab^	80.00 ± 1.73^ab^	98.67 ± 2.08^de^	243.33 ± 1.15^cd^	2056.67 ± 25.17^ab^	31.00 ± 0.00^ac^	13.33 ± 0.58^a^	7.10 ± 0.10^a^
BS-114-BS-120	4.33 ± 0.06^bc^	10.33 ± 0.40^a^	1.33 ± 0.58^a^	16.00 ± 2.00^bc^	3.33 ± 0.58^b^	0.24 ± 0.02^ab^	76.33 ± 2.31^b^	91.00 ± 1.73^e^	236.33 ± 8.08^d^	1993.33 ± 56.86^ab^	31.33 ± 2.08^ab^	11.33 ± 0.58^ab^	7.13 ± 0.06^a^
PPW-26	4.23 ± 0.15^c^	10.23 ± 0.64^a^	1.00 ± 0.00^a^	12.33 ± 4.16^c^	13.33 ± 8.62^a^	0.22 ± 0.01^b^	63.33 ± 3.21^c^	113.33 ± 9.29^bc^	259.33 ± 14.43^abc^	1963.33 ± 75.72^ab^	27.33 ± 3.21^bc^	9.33 ± 2.31^b^	6.93 ± 0.55^a^
CS	4.57 ± 0.15^ab^	10.77 ± 0.55^a^	1.00 ± 0.00^a^	22.00 ± 1.00^ab^	3.67 ± 0.58^ab^	0.25 ± 0.01^ab^	64.33 ± 1.53^c^	105.67 ± 3.06^cd^	250.67 ± 5.69^ad^	1930.00 ± 43.59^b^	32.33 ± 0.58^a^	14.67 ± 1.15^a^	6.87 ± 0.15^a^

Values represent the mean ± SD (n= 3). OM, organic matter; C: N, carbon-to-nitrogen ratio; NO_2_^-^, nitrite; NO_3_^--^N, nitrate nitrogen; NH_3_, ammonia; TKN, total Kjeldahl nitrogen; P-HCO_3_^-^, bicarbonate-extractable phosphorus; K^+^, potassium; Mg^2+^, magnesium; Ca^2+^, calcium; Na^+^, sodium; S, sulfur. Different superscript letters within the same column indicate significant differences among treatments according to one-way ANOVA followed by Tukey’s *post-hoc* test (P < 0.05).

## 4 Discussion

This study takes an integrated approach to evaluate different bacterial-based treatments for improving crop growth and performance in common bean as a model crop. Different formulations, including live bacterial cells, cell-free extracts, consortia, and combinations with reduced fertilizer inputs, were evaluated. Treatments were assessed across crop developmental stages and experimental conditions and at different biological response levels. Overall, treatment-dependent effects were observed in seed germination, seedling vigor, plant growth, reproductive development, and physiology, with responses varying by strain, formulation, and plant developmental stage. This work moves beyond a single application strategy or response endpoint by examining multiple complementary dimensions of bacterial treatment performance within one integrated study, providing a broader view of both their practical versatility and the factors that shape their efficacy. This evaluation provides a more comprehensive framework for understanding the potential and limitations of bacterial-based biostimulants and demonstrates that treatment performance cannot be reliably inferred from isolated early-stage responses or individual crop parameters alone. Instead, efficacy is shaped by multiple interacting factors, such as the continuity of crop responses across development and the persistence of treatment effects under agronomically relevant conditions.

Biopriming of seeds with bacterial cultures showed accelerated early germination processes (**Figure 1A**), although all treatments achieved similar germination percentages after additional incubation. Similar responses have been reported with PGPRs accelerating the timing of radicle emergence in seeds, without altering the final germination percentages (Rozier et al., 2019). Accelerated early germination processes may reflect enhanced mobilization of seed reserves. Consistent with this, genomic annotation of the tested bacterial strains identified hydrolytic enzyme-related genes including phytases and amylases (Kaddoura et al., 2026). These can mobilize seed reserves by hydrolyzing starch and phytate, releasing soluble sugars and inorganic phosphate required for early physiological processes during germination (Al Hijab et al., 2024). Bacterial treatments also improved seedling vigor parameters. The treatments enhanced root system development during early seedling growth through coordinated increases in root length and biomass, indicating structural development rather than simple elongation (**Figures 1B–D**). Notably, BS-114 displayed more pronounced SRL values compared with PPW-26. Bacterial treatments induced distinct root architectural responses through which similar improvements in seedling vigor were achieved (**Figures 1B, G**). BS-114 seedlings displayed a stronger elongation-oriented response, whereas PPW-26 exhibited a more balanced pattern between elongation and structural development. Distinct root architectural strategies suggest early modulation of developmental and hormonal regulation pathways. Similar observations were reported in soybean, where inoculation with *B. subtilis* promoted root development and altered its architecture (Moraes et al., 2025). These responses are consistent with the previously characterized ability of the tested strains to produce indole-related compounds, which may contribute to modulation of root developmental processes (Kaddoura et al., 2026). Moreover, culture-based recovery, followed by validation against the expected MLST allele sequences, provided strong evidence that BS-114 internally colonized common bean roots during early seedling establishment. However, the persistence of BS-114 beyond this early developmental stage was not evaluated, and colonization was not confirmed for BS-120 or PPW-26. Importantly, the absence of confirmed internal colonization does not rule out plant growth-promoting activity, as beneficial bacteria may still act through root-surface interactions. Endophytic colonization may provide additional routes of interaction within plant tissues; however, the present study design cannot determine whether the effects of BS-114 were dependent on internal colonization or root-surface activity. Together, these findings indicate that bacterial treatments influenced early development and root architecture during seedling establishment.

Under controlled greenhouse conditions, treatment effects were expressed differently across vegetative and reproductive stages. Plant height showed a stronger response than stem diameter, while total fresh mass did not increase consistently across bacterial treatments (**Figures 2D, E**). This suggests that vegetative responses are trait-specific and cannot be interpreted simply as overall growth stimulation. Additionally, the LB control provided important context for separating nutrient-driven growth from developmental performance associated with the bacterial treatments. Although LB promoted vegetative fresh mass, this response was not accompanied by proportional improvements in reproductive development. Similarly, NPK enhanced some vegetative traits but did not consistently produce the strongest reproductive outcomes.

Together these observations indicate that nutrient-driven vegetative responses were not sufficient to sustain reproductive performance and that treatment effects extended beyond nutrient availability alone. Bacterial treatment effects were more strongly evident at reproductive development stage. BS-114 and to a lesser extent PPW-26, promoted earlier reproductive progression, as reflected by the earlier pod formation observed at week 6 and the higher reproductive structure formation during the initial reproductive stages (**Supplementary Figure 1**). This suggests that bacterial treatments advanced the onset of reproductive development, with early differences maintained through later stages. These patterns of increased reproductive structures and reduced time to onset are consistent with other PGPR reports (Kumari et al., 2015; Lyu et al., 2022). However, early increases in reproductive structures were not fully retained, as reflected by the differences between immature and mature pod counts (**Figures 3C, D**). This likely reflects reproductive attrition during development, yet these early advantages continued to shape final yield outcomes (**Figures 3E, F**). Yield responses were reflected in multiple reproductive components rather than a single trait. Increases in cumulative pod fresh mass under bacterial treatments were accompanied by increases in both pod number and average pod fresh mass, indicating that reproductive performance depended on continuity of improvements from pod initiation to filling. Despite losses during later developmental stages, bacterial treatments maintained greater reproductive output through to maturity. Overall, this suggests that bacterial treatments influenced reproductive timing, developmental continuity, and final yield formation rather than simply enhancing vegetative growth.

Greenhouse evaluation of the different bacterial formulations revealed strain- and formulation-dependent vegetative responses (**Figures 4A–C**). In particular, the stronger increase in stem thickness under BS-114E relative to its live-cell counterpart suggests that the effect was not solely dependent on viable cells and may have been associated with extracellular components in the extract. These observations indicated that treatment responses were influenced by bacterial formulation. Additionally, combination treatments showed responses comparable to the NPK control, without consistently exceeding its effects, suggesting that the observed plant responses were not solely attributable to nutrient input. Reproductive responses were more pronounced than vegetative responses and varied across developmental stages. However, early responses at reproductive initiation were not retained uniformly across treatments. Differences in buds and flower counts suggested variation in the progression of reproductive development (**Figures 5A, B**). BS-114E showed the strongest continuity from bud formation to pod number and cumulative pod mass while NPK and CS.NPK50 also performed strongly at final yield, as reflected in total pod weight and average pod mass (**Figures 5C–E**). Previous characterization of BS-114 showed indole-related compound production and biosurfactant activity (Kaddoura et al., 2026). Together with the current observations, this suggests that soluble bacterial metabolites may have contributed to the overall positive effects observed across both vegetative and reproductive traits. In contrast, PPW-26E, despite its strong effect at the flowering stage, showed comparatively moderate performance at the pod and yield levels. Together, these observations indicate that early reproductive responses were not sufficient predictors of final yield outcomes, as reproductive performance depended on the progression and retention of reproductive structures through later developmental stages. These patterns were consistent with those observed in GH1.

Physiological responses were primarily expressed through increases in photosynthetic rate and stomatal conductance, whereas intercellular CO_2_ concentration and transpiration remained comparatively stable across treatments. Although PPW-26L, BS-114L, and CS.NPK50 showed the strongest physiological responses, these improvements did not consistently correspond to reproductive outcomes. For example, PPW-26L exhibited the highest photosynthetic rate, whereas BS-114E achieved greater reproductive performance despite more moderate physiological responses (**Figure 4D**, 5C–E). This contrast suggests that enhanced carbon assimilation alone was insufficient to explain differences in productivity. Within this context, source–sink relationships, particularly the balance between photosynthetic carbon supply and reproductive demand, may provide a useful framework for interpreting differences in the retention and development of reproductive structures (Soltani et al., 2019). Similarly, the stronger physiological responses under PPW-26L and BS-114L, compared with the reproductive performance of their corresponding extracts, suggest that live cells may be more closely associated with leaf-level physiological activity, whereas cell-free extracts may influence sink development or reproductive allocation. Although not all differences were significant, this pattern was observed across both strains. Notably, the bacterial consortium combined with half the fertilizer dose (CS.NPK50) sustained vegetative growth and final yield comparable to the full-dose consortium treatment (CS.NPK100) and the NPK control, while showing stronger physiological responses than either treatment, particularly in photosynthetic rate and stomatal conductance. This suggests that consortium application may help maintain productivity under reduced fertilizer input, supporting its potential role in fertilizer-reduction strategies. This is consistent with previous findings in corn, where co-inoculation with *B. subtilis* and *Azospirillum brasilense* under reduced nitrogen fertilization enhanced leaf gas exchange parameters, improved plant nitrogen- and water-use efficiency, and supported greater biomass and grain yield (Galindo et al., 2024).

The carbon fixation-related gene *rbcL*, which encodes the large catalytic subunit of RuBisCO, showed only minor changes across treatments despite the increases observed in photosynthetic performance. Notably, BS-114L exhibited higher *rbcL* expression, whereas PPW-26L showed higher photosynthetic rates, indicating that transcriptional changes in carbon fixation did not correspond directly with gas exchange measurements or plant performance. The limited alignment between *rbcL* expression and photosynthetic performance in the live-bacteria treatments suggests that increased CO_2_ assimilation was not driven by *rbcL* expression alone. *Bacillus* spp. have been previously reported to enhance photosynthetic performance and modulate photosynthesis-related processes, including carbon metabolism and RuBisCO-associated activity, although direct evidence linking beneficial bacterial inoculation to *rbcL* transcription remains limited (Wang et al., 2022). Similarly, inoculation with an endophytic *Pseudomonas oryzihabitans* CB24 was described to enhance carbon assimilation and RuBisCO activity in *Pelargonium graveolens* while upregulating several photosynthesis-related genes involved in light harvesting and electron transport (Deepa et al., 2025). In common bean, proteomic analysis further showed differential regulation of RuBisCO and proteins involved in its activation and assembly (Zadražnik et al., 2017). Together, these findings suggest that improved photosynthetic performance may involve broader physiological mechanisms and post-transcriptional regulation of RuBisCO rather than changes in *rbcL* expression alone. The nitrogen fixation-related gene *nifH*, which encodes the dinitrogenase reductase subunit of nitrogenase, was strongly upregulated across treatments, with the highest expression observed under BS-114L, followed by PPW-26L. Consistent with this, increased nodulation was observed (**Figure 2A**), indicating enhanced associations with nitrogen-fixing microorganisms. However, *nifH* expression and nodule formation reflect the presence of nitrogen fixation potential rather than actual nitrogen fixation rates, which are influenced by multiple environmental and physiological factors (Singleton and Stockinger, 1983; McCormick, 2018). A similar distinction was observed in soybean, where co-inoculation with *Bradyrhizobium japonicum* and *B. aryabhattai* enhanced nodule nitrogenase activity despite relatively low rhizosphere *nif* gene abundance, indicating that *nif*-related molecular responses did not directly reflect functional nitrogen fixation (Xing et al., 2022). In this study, despite these increases, *nifH* expression alone was insufficient to explain differences in physiological performance and yield outcomes, suggesting that broader plant–microbe and physiological processes contributed to the final response. Bacterial treatments also modulated auxin- and cytokinin-related gene expression across tissues. Auxin-related genes (*AUX* and *AUX/IAA*) were consistently upregulated, suggesting enhanced auxin transport and signaling activity. In contrast, cytokinin-related genes displayed clear tissue-specific regulation, with increased IPT3 expression in roots and reduced expression of *IPT3* and *LOG* in leaves, indicating redistribution of hormonal regulation across tissues. Reduced *PVCK* expression is also consistent with reduced cytokinin degradation, further supporting modulation of cytokinin-related pathways. This pattern reflects a coordinated rebalancing of auxin–cytokinin signaling, indicating that hormonal activity was redistributed rather than uniformly elevated across the plant. Such coordinated regulation is consistent with established auxin–cytokinin crosstalk, in which cytokinin can modify auxin transport and signaling to regulate plant development (Street et al., 2016). Importantly, hormone-related regulation has also been observed following microbial inoculation, with bacterial consortia in Arabidopsis differentially modulating auxin-, cytokinin-, and jasmonate-responsive signaling, while a Bacillus-containing consortium promoted flowering and reproductive output (Guerra et al., 2026). Taken together, these responses indicate that both hormonal pathways were actively regulated in parallel, with their relative balance likely contributing to the developmental outcomes observed throughout the study rather than being driven by either pathway independently. Overall, hormone-related gene expression aligned more closely with the observed vegetative and reproductive responses than carbon- or nitrogen-related gene expression. These findings suggest that treatment effects were associated with regulation of developmental processes rather than uniform enhancement of growth.

Field trials conducted under agronomic conditions showed consistent increases in yield across all treatments relative to the NC control. Among treatments, BS-114+BS-120 and NPK exhibited the highest yields, followed by CB and PPW-26, whereas BS-114 and BS-120 showed comparatively smaller gains. The best-performing bacterial treatments showed yield responses comparable to both NPK and the commercial biostimulant (CB). These results indicate that positive yield responses to selected bacterial treatments persisted under field conditions. Although differences were not statistically significant, yield responses consistently favored bacterial treatments over the NC control.

Consortia-based treatments generally outperformed single isolates; specifically, the *Bacillus* consortium (BS-114+BS-120) closely matched NPK performance while the *Bacillus*–*Pseudomonas* consortium showed smaller improvements, suggesting that compatibility among strains may influence field performance. This aligns with meta-analytic evidence showing that microbial consortia generally outperform single-strain inoculants, with functional complementarity and compatibility among constituent strains contributing to their efficacy (Liu et al., 2023).

These shifts in microbial community composition were accompanied by subtle but structured changes within taxa associated with nutrient cycling and soil functionality. At the phylum level, communities across all treatments were dominated by Verrucomicrobia, Actinobacteria, Proteobacteria, and Firmicutes, consistent with previously reported core microbial taxa associated with *Phaseolus vulgaris* rhizosphere soils (Stopnisek and Shade, 2021). Relative to NC, Verrucomicrobia decreased most strongly under BS-114, followed by PPW-26 and CS. Verrucomicrobia, commonly associated with oligotrophic conditions and carbon turnover, are often reported to decrease with increasing nutrient availability (Navarrete et al., 2015), suggesting that this reduction may reflect treatment-associated improvements in soil nutrient availability. In contrast, Actinobacteria increased across all bacterial treatments, with the most notable increases observed under BS-114+BS-120, PPW-26, and CS, whereas Firmicutes increased mainly under BS-120 and CS. These increases are relevant as both Actinobacteria and Firmicutes are widely recognized for their roles in organic matter decomposition, nutrient mineralization, and production of antimicrobial compounds (Bhatti et al., 2017; Anderson et al., 2018), contributing to soil fertility and potential disease suppression (Cha et al., 2015). Moreover, the relative abundance of *Bradyrhizobium* slightly increased under BS-114+BS-120, PPW-26, and CS. As a well-established nitrogen-fixing genus in legume systems, this increase is consistent with enhanced nitrogen-cycling potential and plant-microbe interactions. Previous studies have shown that co-inoculation of *Bradyrhizobium* with PGPRs, including *Bacillus* species, can enhance nodulation, nitrogen accumulation, and N-fixation-related traits in legumes (da Costa Neto et al., 2024). The presence of *Bacillus* species across treatment groups is also relevant because members of this genus are associated with plant growth promotion, nutrient solubilization, and biocontrol activity (Miljaković et al., 2020). Slight increases in *Candidatus Nitrocosmicus*, particularly under NPK and CS, suggest increased nitrification potential (Lee et al., 2024), consistent with observed increases in nitrate levels. Similarly, the enrichment of *Pseudarthrobacter* in BS-114+BS-120, PPW-26, and CS suggests enhanced organic matter turnover and nutrient mobilization, as members of this genus have been associated with phosphorus solubilization and stress tolerance in soil systems (Ham et al., 2022).

Consistent with these microbial shifts, soil nutrient analysis revealed significant differences in several nutrient and soil fertility parameters, whereas C;N ratio, nitrite, and pH remained comparatively stable across treatments. Treatment-level alignments with soil nutrient changes were most evident in treatments that also showed increases in taxa associated with nutrient cycling and organic matter turnover. Differences were particularly evident for nitrogen forms, bicarbonate-extractable phosphorus, and sulfur, although no consistent directional pattern was observed across bacterial treatments. These patterns suggest. That the observed microbial shifts were associated with changes in specific nutrient pools and functional processes, including organic matter turnover, phosphorus mobilization, and nitrogen transformations. Higher phosphorus and sulfur levels under select treatments indicate improved availability of key macronutrients involved in plant development. Together, these shifts in nutrient availability indicate that treatments influenced soil nutrient dynamics rather than overall soil fertility. Despite these microbial and nutrient shifts, microbial community structure remained largely conserved across treatments, with no clear separation observed in beta diversity and only minor changes in alpha diversity. This suggests that bacterial treatment application primarily influenced existing microbial processes, particularly those related to nutrient cycling, rather than substantially restructuring the soil microbiome, a pattern consistent with short-term field experiments (Reese et al., 2018). .

## 5 Conclusion

This study evaluated previously characterized bacterial endophyte-based treatments with plant growth-promoting traits for their effects on yellow bean performance across early development, greenhouse production, and field conditions. Overall, crop responses to the bacterial treatments were influenced by plant developmental stage, strain-specific functional traits, and formulation, with effects varying among live bacterial cells, cell-free extracts, individual strains, and consortia. Moreover, bacterial treatments influenced early germination, root development, and physiological activity; however, these responses did not uniformly translate into increased biomass or yield. Instead, treatment success was more consistently associated with the maintenance of reproductive development throughout plant growth, most notably under *Bacillus*-based treatments, indicating that developmental continuity was more important than the magnitude of individual responses. While physiological, molecular, and microbial responses were repeatedly observed, no single response fully explained plant performance. However, hormone-associated responses, particularly the coordinated modulation of auxin-cytokinin pathways, provided the strongest mechanistic link to the observed improvements, especially during reproductive development. Field studies showed that bacterial treatments can be effective in real agricultural settings, with certain treatments achieving yield gains comparable to conventional fertilizer and commercial biostimulant inputs. These improvements were also accompanied by treatment-specific rhizosphere microbial and nutrient shifts, suggesting that even short-term bacterial applications can influence existing soil processes without necessarily inducing major restructuring of the soil microbiome. Additionally, the performance of combination treatments further suggests that bacterial treatments may have potential not only as alternatives to conventional inputs, but also as complements to fertilizer-based strategies. Collectively, these findings highlight that treatment efficacy cannot be inferred solely from individual responses, such as vegetative growth, carbon assimilation, or nitrogen-related activity, which were not reliable predictors of field performance when considered individually. Instead, successful microbial interventions should be comprehensively evaluated through the coordination of developmental, physiological, molecular, and microbial responses across plant stages, which may support more reliable selection and increased adoption of bacterial treatments for sustainable crop production.

## Data Availability

The datasets presented in this study can be found in online repositories. The names of the repository/repositories and accession number(s) can be found in the article/**Supplementary Material**.
