# Supplementary material for: Greenhouse and field evaluation of bacterial endophytes from Cannabis sativa and Chelidonium majus reveals beneficial stage-dependent growth and yield responses in common bean
Source: Front Plant Sci. 2026 Aug 27;17:1922467. doi: 10.3389/fpls.2026.1922467 (PMC13560875; doi:10.3389/fpls.2026.1922467)
Supplement: Supplementary file 1 [file DataSheet1.docx]

Supplementary Material

**Supplementary Table S1:** Summary of cycling conditions used in the study.

| **Step** | | **Temperature** | **Time** |
| --- | --- | --- | --- |
| Primary denaturation | | 95°C | 1 min |
| 40 cycles | Denaturation | 95°C | 5 sec |
|  | Annealing/Extension | 55°C | 10 sec |
| Melt curve | | 65–95 °C | 5 sec /0.5 °C increment |

**Supplementary Table S2:** Primer sequences used for RT-PCR analysis in yellow beans.

| **Gene** | **Functional Annotation** |  | **Primer Sequence (5’-3’)** | **Reference** |
| --- | --- | --- | --- | --- |
| Act11 | Actin-11  (Reference gene) | F | TGCATACGTTGGTGATGAGG | (Porteous-Álvarez et al., 2020) |
|  |  | R | AGCCTTGGGGTTAAGAGGAG |  |
| NifH | Nitrogenase iron protein | F | GGTTGTGACCCGAAAGCTGA | (Bürgmann et al., 2003) |
|  |  | R | GCGTACATGGCCATCATCTC |  |
| AUX6 | Auxin transporter-like protein 2 | F | CGTCGCCGTCTATTGGGCTT | (Nanjareddy et al., 2024) |
|  |  | R | ACCGTGCAAGTGCCCTCAAA |  |
| AUX/IAA | Auxin-responsive protein | F | GCATCCACTCATCGGAATCT | (Ekinci et al., 2023) |
|  |  | R | GGAACCCGAACATTTTGAAC |  |
| PVCK01 | Cytokinin oxidase | F | AGGCGAAACCTTAGTCTG | (Zhang et al., 2023) |
|  |  | R | AGCCACTCTGCGTCTCTA |  |
| AIPT3 | Adenylate isopentenyltransferase 3 | F | CAGCCTCTGAAAAACGTTCC | (Ekinci et al., 2023) |
|  |  | R | TCTCCCAGAAGTGGAATTGG |  |
| Log1/Log1-like | Cytokinin riboside 5′-monophosphate phosphoribohydrolase (LOG family) | F | TGGCAGTAGTCCAGGGAACA | This study |
|  |  | R | CATCAAACCAATGCTGCCCC |  |

**Supplementary Table S3: MLST allelic profiles of BS-114 and BS-116 and housekeeping genes selected for strain-level confirmation**

| **Strain** | **MLST** | **Allelic Profile** | **Reference** |
| --- | --- | --- | --- |
| BS-114 | Novel | glpF(1), **ilvD(48**), **pta(55)**, **purH(49)**, **pycA(34)**, rpoD(1), tpiA(1) | (Kaddoura et al., 2026) |
| BS-116 | ST-105 | glpF(3), **ilvD(64)**, **pta(44)**, **purH(66)**, **pycA(2)**, rpoD(3), tpiA(3) | (Kaddoura et al., 2026) |

*Housekeeping genes selected for strain-level confirmation in this study are shown in bold.

**Supplementary Table S4: Primer sequences used for housekeeping-gene amplification and strain-level confirmation**

| **Primer** | **Sequence (5’–3’)** | **Expected Amplicon Size (bp)** | **Reference** |
| --- | --- | --- | --- |
| pycA-F | AAATCAGARGCGAAAGC | 545 bp | (Le et al., 2019) |
| pycA-R | CCTGAGCGGTAAGCCAT |  |  |
| pta-F | ATACATATGAAGGSATGGAAGA | 610 bp | (Le et al., 2019) |
| pta-R | TAGCCGATGTTTCCTGCT |  |  |
| purH-F | TTTGAGAAAAAACAATCGCT | 568 bp | (Le et al., 2019) |
| purH-R | TCGGCTCCCTTTTCGTCGG |  |  |
| ilvD-F | ATGAGATATTCGCTGCC | 622 bp | (Le et al., 2019) |
| ilvD-R | CTTCGTTAATGCGTTCTAAAGAG |  |  |

**Supplementary Figure S1:** Vegetative growth parameters of treated yellow bean plants at week 6. NC, non-inoculated control; NPK, chemical control; CB, fermented algae-based commercial biostimulant control; M, LB media control; BS-114, *Bacillus subtilis* strain 114; PPW-26, *Pseudomonas wadenswilerensis* strain 26. Average early immature pod count per plant (week 6). Values represent mean ±SD (n= 5). Different letters indicate significant difference relative to the control according to one-way ANOVA followed by Dunnett’s *post-hoc* test (P < 0.05).
